# Psychological Intervention in Traumatic Brain Injury Patients

**DOI:** 10.1155/2019/6937832

**Published:** 2019-05-02

**Authors:** Lizzette Gómez-de-Regil, Damaris F. Estrella-Castillo, Julio Vega-Cauich

**Affiliations:** ^1^Hospital Regional de Alta Especialidad de la Península de Yucatán, Calle 7, No. 433 por 20 y 22, Fraccionamiento Altabrisa, Mérida, Yucatán, 97130, Mexico; ^2^Autonomous University of Yucatan, School of Rehabilitation, Avenida Itzáes No. 498 x 59 y 59A, Colonia Centro, Mérida, Yucatán, C.P. 97000, Mexico; ^3^Foco Rojo-Centro de Psicología Aplicada, Calle 47, No. 506 por 62 y 64, Colonia Centro, Mérida, Yucatán, C.P. 97000, Mexico

## Abstract

**Objective:**

To provide a brief and comprehensive summary of recent research regarding psychological interventions for patients surviving a traumatic brain injury.

**Methods:**

A bibliographical search was performed in PubMed, Cochrane Library, PsycNET, Scopus, ResearchGate, and Google Scholar online databases. Analysis included distribution by year of publication, age stage of participants (paediatric, adult), location of the research team, study design, type of intervention, and main outcome variables.

**Results:**

The initial search eliciting 1541 citations was reduced to 62 relevant papers. Most publications had adult samples (88.7%). The United States outstands as the country with more research (58.1%); Latin America countries provided no results. Cognitive behavioural therapy (CBT) was the most widely used approach for treatment of (sub)clinical mental disturbances (41.9%). Neuropsychological interventions were scarce (4.8%). Outcome measures included psychiatric disorders (e.g., posttraumatic stress disorder (PTSD), depression, and anxiety) (37.1%), postconcussive symptoms (16.1%), cognitive and functional deficits (48.1%), and social and psychological dimensions (62.9%).

**Conclusions:**

CBT outstands as the preferred therapeutic approach for treating behavioural and emotional disturbances. Also, other related therapies such as dialectical behaviour, mindfulness, and acceptance and commitment therapies have been proposed, and probably in the years to come, more literature regarding their effectiveness will be available. On the other hand, evidence showed that interventions from the field of neuropsychology are minimal if compared with its contribution to assessment. Future research should be aimed at performing studies on more diverse populations (e.g., nonmilitary communities and paediatric and Latin American populations) and at controlling designs to examine the therapeutic efficacy of psychotherapeutic and neurocognitive rehabilitation interventions and compare amelioration by injury severity, age of patients, and clinical profile, in the hopes of creating better guidelines for practitioners.

## 1. Introduction

Traumatic brain injury (TBI) is a disruption in normal brain function caused by external mechanical force, such as rapid acceleration or deceleration, a bump or jolt to the head, or penetration by a projectile. As an acquired brain injury (i.e., postnatal brain damage), TBI is differentiated from nontraumatic brain injuries not involving an impact from external forces (e.g., those caused by strokes and infections). Considering symptom severity and duration (loss of consciousness, posttraumatic amnesia, and memory and motor deficits), TBI can be classified as concussion, mild, moderate, or severe [1, 2].

Someone with TBI, even if medically stable, is likely to experience subsequent symptoms ranging from physical (headache, fatigue, and visual/auditory sensitivity) to cognitive (deficits in memory, attention, concentration, and executive function) and emotional (depression, anxiety) symptoms [1]. Various treatment modalities have been proposed and tested, from medical/surgical to behavioural/cognitive methods (see reviews [3–5]). Addressing impairments that cut across multiple disciplines requires assessment and rehabilitation following an interdisciplinary model with a team of experts on physical medicine and rehabilitation, speech-language pathology, social work, and (neuro)psychology, among others [6].

Current TBI therapies include pharmacotherapy, psychotherapy, and cognitive rehabilitation. However, psychological and emotional issues often remain overlooked even when physical, behavioural, and cognitive symptoms are treated [7]. Psychology has a long history of research and practice on neuropsychological assessment of TBI patients, and there is a growing interest in designing, testing, and providing suitable psychological interventions.

Psychology has contributed to TBI patient care mainly from a neuropsychological perspective. Neuropsychology is a hybrid science in which psychology, psychiatry, and neurology converge in the study of connections between the brain and behaviour. Assessment has been its main role. This is done through techniques and instruments aimed at evaluating patient neurocognitive, behavioural, and emotional strengths and weaknesses and interpreting their link to brain anatomy and function [8]. Beyond clinical diagnosis for treatment planning and progress, neuropsychological assessment has also become a core aspect of decision-making regarding function and disability in legal [9], labour [10], and sports [11, 12] contexts.

As a result of TBI, cognitive (e.g., deficits in attention, memory, and executive function) and behavioural (e.g., aggression, poor impulse control, irritability, anhedonia, or apathy) symptoms may occur and psychiatric/affective disorders may initiate or worsen [13]. Beyond neuropsychological assessment, psychologists have also worked intensively on the design, implementation, and testing of post-TBI interventions. Psychology has aided in the cognitive rehabilitation of TBI patients [14, 15], as well as in helping them to manage the emotional impact of this condition through psychotherapy [16] or psychoeducational programs [15]. Family interventions are another technique applied to TBI survivors since the condition can adversely impact relatives, who often play a critical supporting role in the patient recovery process [17].

Given their particular health conditions, TBI survivors may require professional psychological support to deal with both cognitive and emotional challenges. This review is aimed at providing a brief and comprehensive summary of recent research on psychological interventions in TBI survivors that are potentially of interest to professionals working with this population.

## 2. Method

A bibliographical search was performed in the PubMed, PsycNET, Web of Science, Scopus, Cochrane Library, and Google Scholar databases. The terms “traumatic brain injury” and “TBI” were entered in combination with “psychology”, “neuropsychology”, “psychoeducation”, and “psychotherapy”. Filters were applied to retrieve only articles published in English during the decade prior to the search (2008 to July 2018). Online resources were accessed on 25 to 27 July 2018. Publication relevance was verified based on the study objective. Citations for publications other than research articles (e.g., commentary, erratum, and editorials) were excluded, as were articles reporting (systematic) reviews and/or meta-analyses. Abstracts were used to make a further cull of publications reporting research not clearly related to patients with TBI and/or not focused on psychological intervention. Once a final reference list was generated, a series of data were collected on each article: year of publication, participant age stage (paediatric, adult), research team location, study design, intervention type, and main outcome variables. All three authors worked together during the bibliographic research procedure, and discrepancies were minimal.

## 3. Results

**Figure 1:**
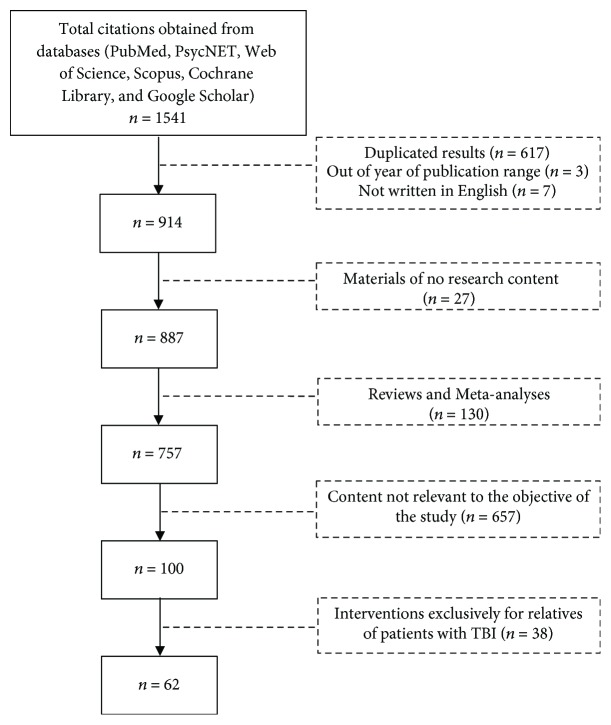
Study flow diagram.

The initial search from the six selected databases produced 1,541 citations, of which 617 were duplicates, three were not within the specified publication year range, and seven were not in English. After applying the exclusion criteria and reviewing the available abstracts, the list was reduced to 62 relevant publications (Figure 1).

Classification by participant age stage, year, location, and study design showed that most of the studies have been done using adult samples (*n* = 55, 88.7%), with participants aged 16 to 73 (Table 1). Only a small portion (*n* = 7, 11.3%) involved paediatric samples; participants were 4 to 18 years old, and the studies were done in the United States (*n* = 5) and Italy (*n* = 2). The number of relevant publications increased notably during the last three years of the publication time range and accounted for 41.9% (*n* = 26) of the results; no results were found for the year 2010. Most of the studies are from the United States (*n* = 36, 58.1%) and were done with adult samples (*n* = 31, 86.1%). Over half the studies performed with adults (54.8%) included veterans or active-duty service members, either exclusively (*n* = 15) or in combination with civilians (*n* = 2). The research from all the other countries was done with nonmilitary populations. Fifteen of the remaining studies are from Europe and were done in Finland, France, Italy, the Netherlands, Norway, Slovenia, Switzerland, and the United Kingdom; two studies are from Asia, one from India, and the other from Malaysia.

In terms of study design, four protocols describe 2-group (*n* = 3) and 3-group (*n* = 1) randomized control trials; these were done in the United States, Australia, Norway, and Finland. The case-study reports only involved adults (8 civil cases and 3 veterans). One qualitative study analysed patient interaction content to provide feedback to therapists and improve their performance. One-group quasiexperimental designs (*n* = 13) were mostly tested in community samples (*n* = 7), and four of these were pilot studies. Most of the randomized control trials were two-armed (*n* = 27), but there were also 3-armed (*n* = 4) and four-armed (*n* = 1) studies; two were pilot studies and three included wait list control groups. Only one two-armed study was a nonrandomized trial.

Two types of interventions were used: cognitive behavioural therapy (known as CBT) was the intervention technique of choice (*n* = 26, 41.9%), followed by psychoeducation (*n* = 16, 25.8%). Very few publications addressed neuropsychological interventions in TBI patients (*n* = 3, 4.8%). Outcome measures were diverse, and most studies included various domains, such as psychiatric disorders (e.g., PTSD, depression, and anxiety) (*n* = 23, 37.1%), postconcussive symptoms (*n* = 10, 16.1%), cognitive and functional deficits (*n* = 30, 48.1%), and social and psychological aspects (*n* = 39, 62.9%) (Table 2).

Of note, not all the reported interventions were done following standard, face-to-face techniques. Limited access to psychological services has fuelled increasing interest in implementing technology to broaden intervention options. Some studies involved telephone-based [18–21] or computer-based [22, 23] interventions, while others employed diverse technologies such web-based programs [24, 25], mobile applications [26], videogames [27], and virtual reality [28].

## 4. Discussion

This review is a brief, comprehensive overview of scientific manuscripts reporting on psychological treatments applied to TBI survivors with the purpose of helping them to directly or indirectly overcome cognitive and emotional issues linked to their physical condition.

Once a TBI patient is physically stable, subsequent cognitive, emotional, behavioural, and social difficulties may manifest, hindering engagement with treatment and daily activities. Managing these challenges requires a comprehensive neuropsychological treatment approach. As the most widely used psychotherapeutic approach, CBT is built on the assumption that cognitions (i.e., thoughts) strongly affect behaviours, but, through awareness, can be quantified and controlled. In other words, a person can attain behavioural changes through acknowledgment and control of preceding cognitions. Application of CBT for TBI patients has been aimed at reducing anger, depression, anxiety, and PTSD symptoms and at improving coping, with promising results [29, 30]. However, adaptations are still needed for this population to improve intervention efficacy and allow replication [31]. If the aim of a multidisciplinary team is to achieve the best possible outcome, the medical professionals involved need information on the aims and techniques of psychotherapy whereas the psychotherapists need to understand the disorder's medical characteristics. Psychotherapy with TBI patients can be challenging and frustrating at times but is worth attempting since it can be very rewarding for both the survivor and therapist [30].

Research on psychological interventions in TBI patients has grown over the last decade and boomed during the last three years. The United States is the apparent leader in this research area since TBIs have been acknowledged as an important public health issue. Estimates from the United States indicate that TBIs annually account for approximately 2.5 million emergency room visits, hospitalizations, and deaths nationwide; however, this does not include sufferers who did not receive medical care, had outpatient or office-based visits, or were treated at a federal facility (e.g., active-duty military members and veterans). Those who have served in the United States military are at significant risk for TBI; for instance, an estimated 4.2% of veterans from the Army, Air Force, Navy, or Marine Corps have been diagnosed with TBI [32]. These particular circumstances may account for the development of various psychological interventions in this country for both research and clinical practices. Research in this area has also been published for populations in Australia, North America, Europe, and Asia. Of note, no results were obtained from Latin America. Researchers and clinicians from this region could benefit greatly from sharing their knowledge on psychological intervention as an element in TBI treatment; initially, this could be done through qualitative and quasi experimental designs requiring little infrastructure.

As observed in a previous review of this area [33], the present results indicated that research in paediatric patients with TBI has been less frequent than that in adults. Although TBIs in children are less frequent, they imply a higher risk of negative impact given that physical and cognitive development are still very much in the process in children. Interventions in paediatric populations also bring additional challenges. For instance, neurocognitive skills are not as fully established as in adults, development is not homogeneous throughout childhood, and the younger the patient the more therapy relies on parents.

Treating patients with psychiatric and neurocognitive symptomatology, which occurs in some TBI cases, can present a unique challenge. Progress in psychotherapy can be significantly hindered by cognitive deficits, and the effectiveness of neurocognitive rehabilitation can be diminished by psychiatric overlay [34]. Ample research is available on neuropsychological assessment of TBI patients, but the present review highlights that publications on neuropsychological interventions are scarce and largely focus on validating specific cognitive rehabilitation techniques. For example, one study in paediatric patients explored the effect of a one-time neuropsychological consultation on postconcussive symptoms [35], while another tested an intervention specifically designed to improve attention, working memory, and executive function [36]. The scientific contribution of neuropsychology to clinical assessment of TBI patients will no doubt continue providing valid and efficient measurements. Neuropsychologists now need to apply themselves to designing, implementing, and testing novel cognitive rehabilitation interventions that, together with neuropsychological evaluations, provide patients with the most adequate treatment.

In terms of outcomes, most of the reviewed studies included mental health variables ranging from symptoms below the clinical threshold to diagnosed mental disorders such as depression, anxiety, or PTSD. Regardless of the approach, be it psychotherapy or neuropsychological rehabilitation, all psychological interventions in TBI patients ideally need to consider outcome variables from both fields, including examination for mental disorders and evaluation of cognitive functioning. The use of scales facilitates assessment of patients for clinical and research purposes, although most outcome scales for TBI are functional measures. As the emotional, cognitive, psychosocial, and health-related quality of life aspects of recovery are increasingly recognised, metrics to assess these domains are becoming essential [37–39]. Clinicians and researchers require reliable, valid measures to comprehensively quantify the level of burden and functional impairment in TBI survivors in the short and long terms. These will improve patient care by allowing proper diagnosis, prompt assignment to rehabilitation, and accurate assessment of intervention impact [37, 39].

In neurological disorders, including TBI, biomarkers play an important role in research and clinical practice by allowing physiological processes to be monitored in health and sickness [40]. Magnetic resonance imaging provides several measurement options that can function as TBI biomarkers, including detection of hemosiderin and white matter abnormalities, assessment of white matter integrity derived from diffusion tensor imaging, and quantitative measurements that directly assess neuroanatomy. Magnetic resonance could also be a useful biomarker in individuals who, having survived TBI, have recovered without neuroimaging signs or neuropsychological effects detected with current methods [41]. Blood biomarkers have also been proposed recently as surrogate markers to improve care quality and reduce diagnosis costs [42]. Evidence-based treatments (i.e., pharmacological or nonpharmacological interventions) of TBI are currently extremely limited, and further research is needed including prospective, longitudinal studies to explore biomarkers along with standard outcome measures [43].

As a final note, TBI severity is an important factor to consider when selecting patients for a specific intervention and for assessing outcome. Because the studies included in this review relied on diverse TBI severity scales (e.g., Glasgow Coma Scale, Barell Matrix, and Abbreviated Injury Scale), some omitted reporting the classification criteria and others did not specify the level of severity and therefore did not analyse this variable.

## 5. Conclusions

This brief overview of recent research on psychological interventions for TBI patients showed that CBT is the preferred therapeutic approach for treating behavioural and emotional disturbances. Other related therapies such as dialectical behaviour, mindfulness, and acceptance and commitment therapies have been proposed, and the literature regarding their effectiveness is sure to grow in the coming years. When compared to its contribution to TBI assessment, neuropsychology is used minimally for interventions. Psychotherapeutic and neurocognitive rehabilitation interventions for TBI patients are challenging for both clinicians and researchers. Future research needs to include more diverse populations (e.g., nonmilitary communities and paediatric and Latin American populations). In addition, it should focus on controlled designs to examine the therapeutic efficacy of psychotherapeutic and neurocognitive rehabilitation interventions and compare amelioration by injury severity, patient age, and clinical profile in the hopes of creating the best practice guidelines for practitioners.

## Figures and Tables

**Table 1:** Distribution of publications on psychological interventions for patients with traumatic brain injury.

	Paediatric sample (age ≤ 18) (*n* = 7)	Adult sample (age ≥ 16) (*n* = 55)	Total (*n* = 62)
Year of publication			
2008	0	1	1
2009	1	3	4
2011	1	4	5
2012	0	9	9
2013	0	5	5
2014	1	6	7
2015	0	5	5
2016	3	7	10
2017	1	10	11
2018	0	5	5
Location			
United States	5	31	36
Australia	0	7	7
Canada	0	2	2
Europe	2	13	15
Asia	0	2	2
Study design			
Protocol	0	4	4
Content analysis	0	1	1
Case study	0	11	11
1-group	3	10	13
2-group	4	24	28
3-group	0	4	4
4-group	0	1	1

**Table 2:** Types of psychological interventions and their main outcome measures.

Type of psychological interventions
Cognitive behavioural therapy (CBT) (*n* = 26) [44–69]
Psychoeducation (*n* = 16) [18–22, 24–26, 53, 70–76]
Cognitive rehabilitation (*n* = 9) [23, 27, 36, 56, 77–81]
Neuropsychological rehabilitation (*n* = 3) [35, 82, 83]
Other (e.g., dialectical behaviour therapy, mindfulness therapy, energy therapy, acceptance and commitment therapy, compassion-focused therapy, and positive psychology) (*n* = 9) [7, 28, 34, 84–89]
Main outcome measures
Mental clinical profile, dysfunctional behaviour, anger, aggressiveness (*n* = 20) [20, 21, 26, 28, 34, 49, 51, 53, 54, 56, 57, 63, 71–74, 83, 84, 89, 90]
Quality of life, life satisfaction, hope, psychological distress (*n* = 19) [21, 27, 28, 47, 52, 57, 58, 60, 64, 65, 68, 72–74, 76, 80, 86–88]
Depression, anxiety (*n* = 15) [7, 20, 46, 50, 54, 55, 58–60, 62, 65, 68, 69, 77, 81]
Daily living, self-care, autonomy, return to work (*n* = 15) [7, 21, 23–26, 55, 57, 70, 72, 76, 77, 80, 81, 89]
Cognitive deficits (e.g., attention, memory, emotion regulation, executive function) (*n* = 15) [27, 34, 36, 45, 56, 57, 67, 69, 72, 73, 78–82]
Postconcussive symptoms (*n* = 10) [22, 24, 25, 35, 64, 66, 72, 74, 76, 89]
PTSD (*n* = 8) [34, 44, 66, 69, 74, 78, 81, 85]

Note: a work may include more than one type of intervention and/or outcome measure.
